# Visceral Adiposity Index Is a Measure of the Likelihood of Developing Depression Among Adults in the United States

**DOI:** 10.3389/fpsyg.2022.772556

**Published:** 2022-03-25

**Authors:** Jun Lei, Yaoyue Luo, Yude Xie, Xiaoju Wang

**Affiliations:** ^1^School of Nursing Hunan University of Chinese Medicine, Changsha, China; ^2^Clinical Nursing Teaching and Research Section, The Second Xiangya Hospital, Central South University, Changsha, China; ^3^College of Integrated Traditional Chinese and Western Medicine, Changsha, China

**Keywords:** NHANES, depressive symptoms, depression, visceral adiposity index (VAI), visceral adiposity

## Abstract

**Background:**

Depression is a serious mental disorder often accompanied by emotional and physiological disorders. Visceral fat index (VAI) is the current standard method in the evaluation of visceral fat deposition. In this study, we explored the association between VAI and depression in the American population using NHANES data.

**Methods:**

A total of 2,577 patients were enrolled for this study. Data were collected through structured questionnaires. Subgroup analysis for the relationship between VAI and depression was evaluated using multivariate regression analysis after adjustment for potential confounding factors.

**Results:**

For every 1 unit increase in VAI, the clinical depression increased by 14% (OR = 1.14, 95% CI: 1.04–1.25). High VAI scores (T3) increased the highest risk of developing depression (OR = 2.32, 95% CI: 1.2–4.47). Subgroup analysis demonstrated a strong and stable association between VAI and the development of depression.

**Conclusion:**

Our study showed that depressive symptoms are associated with a high ratio of visceral adiposity index after controlling confounding factors.

## Background

Depression is a serious mental disorder often accompanied by emotional and physiological disorders (Nguyen et al., 2017; Jackson et al., 2019). Based on the WHO data, there are more than 300 million people with depression worldwide (Puttige Ramesh et al., 2019). In the United States, 16% of people suffer depression at least once in their lifetime (Kessler et al., 2003). Depression usually occurs alongside other chronic diseases. The 15-year recurrence rate of depression in the general population is 35% (Hoen et al., 2010).

Obesity has been implicated in the development of depression (Heo et al., 2006; Rivenes et al., 2009; Luppino et al., 2010; Linde et al., 2011; Haynes et al., 2019). This is evidenced by how the outcome of depression with underlying obesity relies on treatment-seeking behavior (Felitti, 1993). Body mass index (BMI) is a reliable indicator of obesity. In addition, high BMI in adulthood has been linked with depression (Hoen et al., 2010; Mannan et al., 2016). Obesity based on BMI has been linked with the risk of developing depression (Heo et al., 2006; Rivenes et al., 2009; Luppino et al., 2010; Linde et al., 2011; Haynes et al., 2019). However, given that BMI cannot distinguish between visceral fat from fat mass, it is not an accurate measure of obesity (Huang et al., 2019; Yang S. J. et al., 2020; Favre et al., 2021). In related research, the measure of waist circumference (WC), which reflects the level of visceral fat, was found to be positively correlated with depression (Heo et al., 2006). However, just like BMI, WC does not discriminate visceral adipose tissue from abdominal subcutaneous fat (Heo et al., 2006; verson-Rose et al., 2009).

Deposition of visceral fats is associated with high circulating TNF-α and IL-6 and a decrease in insulin sensitivity (Lin et al., 2013; Yang J. et al., 2020). Visceral fats can independently predict the development of depression (Vogelzangs et al., 2008). High visceral fat at baseline, based on CT scan, has been linked with depression (Alshehri et al., 2019). Although imaging techniques such as CT and MRI can directly measure the amount of visceral fat, they cannot be routinely used due to safety and economical limitations. Currently, the visceral adiposity index (VAI) can accurately reflect the accumulation of visceral fats (Amato et al., 2010). Therefore, we explored the relationship between VAI and the development of depression using the National Health and Nutrition Examination Survey (NHANES) data for adults in the United States (US) population.

## Materials and Methods

### Study Design and Data Collection

The National Health and Nutrition Examination Survey (NHANES) was a cross-sectional survey on adults in the US. The study was approved by The Research Ethics Review Board of the National Center for Health Statistics. Data were collected by trained staff through clinical examinations and structured questionnaires. The NHANES data is available at https://www.cdc.gov/nchs/nhanes/default.aspx.

### Study Sample

**FIGURE 1:**
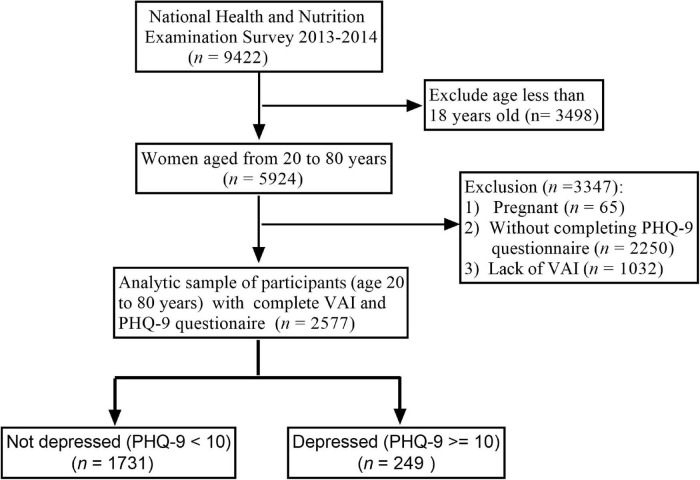
Flowchart of the sample selection from NHANES 2013–2014.

The data were collected from 2013 to 2014 and comprised 9,422 individuals, of which 2,577 were further sampled for interviews. All participants consented to participate in the research. Patients under 18 years of age and those with missing data were excluded from the study. The inclusion and exclusion criteria are summarized in Figure 1.

### Assessment of Primary Variables

#### Scores for Depression

Depression scores within 2 weeks of the interview were calculated using a diagnostic module, the 9-question Patient Health Questionnaire (PHQ-9), as described in earlier studies (2005–2016) (Patel et al., 2019). PHQ-9 scores ≥ 10 were indicative of depression (Jackson et al., 2019).

#### Visceral Adiposity Index Score

The VAI is a gender-specific measure of visceral fat distribution and function based on anthropometric (BMI and WC) and metabolic parameters [high-density lipoprotein cholesterol (HDL-c) and triglycerides] (Amato et al., 2010; Ferguson et al., 2021). The VAI formulae for men and women are shown in Supplementary Table 1. Research shows that the VAI score is directly proportional to the amount of deposited visceral fats (Amato et al., 2010; Ferguson et al., 2021). According to the VAI of individuals in the baseline, three groups (trisection) were categorized as T1: low (0.11–0.92), T2: middle (0.93–1.79), and T3: high (> 1.79).

### Assessment of Study Variables

The potential confounding factors of depression, such as gender, age, race, education level, marital status, diabetes mellitus, family income-to-poverty ratio (PIR), self-reported chronic diseases, WC, BMI, smoking status, dietary intake in a 24-h period, triglycerides, HDL-c, total cholesterol, Vitamin D, glycohemoglobin, low-density lipoprotein cholesterol (LDL-c), and fasting blood glucose, were selected based on previous studies. Triglycerides, total cholesterol, glycohemoglobin, and fasting blood glucose were measured using the NHANES laboratory protocol. Level of education was categorized into several groups, namely, college graduate or above, college or associate (AA) degree, high school graduate, and below 11th grade. Regarding race, the participants were classified into Mexican-American, non-Hispanic black, non-Hispanic white, Hispanic, and others. Marital status included married, living with a partner, never married, divorced, widowed, or separated. Family income-to-poverty ratio was expressed as previously described in which the household income was divided by the poverty threshold. All participants were interviewed two times regarding 24-h feeding habits. The first dietary interviews, which included protein, energy, total sugars, carbohydrate, fibers, and total fat intake, were conducted at the Mobile Examination Center (MEC). Alcohol consumption and smoking were assessed as previously described (Patel et al., 2019). Hypertension was screened based on medical reports or intake of antihypertensive drugs. Hypercholesterolemia was evaluated according to a cholesterol test or previous diagnosis.

### Statistical Analysis

Continuous variables were presented as means, standard errors, percentages, or frequencies. Differences between categorical variables were analyzed using the chi-square test, whereas differences between continuous variables were evaluated using ANOVA or the Man-Whitney *U*-tests based on the nature of the distribution. The association between VAI quartiles and depression was expressed using three models. For Model I, there was no adjustment for confounding factors. In Model II, there were adjustments for age, gender, alcohol drinking, diabetes, smoking status, history of specific diseases, educational status, race, marital status, and family PIR. Categorical variables associated with VAI were converted into continuous variables using the models before analysis. Stratified interaction analyses were performed based on all variables outlined in Table 1. Data were analyzed using Empower-Stats and R software.^1^ A two-sided value of *p* < 0.05 was considered statistically significant.

**TABLE 1:** Baseline characteristics of the cohort (*N* = 2,577).

Characteristics	Visceral adiposity index				*P*-value
	Total (*n* = 2,577)	T1 (*n* = 859)	T2 (*n* = 859)	T3 (*n* = 859)	
Age, year	47.58 ± 18.17	43.85 ± 18.91	48.72 ± 18.01	50.18 ± 16.94	<0.001
BMI, kg/m^2^	28.80 ± 7.19	26.03 ± 6.26	28.90 ± 7.14	31.46 ± 7.09	<0.001
Waist circumference, cm	98.31 ± 16.90	90.24 ± 15.09	98.72 ± 16.22	105.96 ± 15.59	<0.001
**Gender**					0.296
Male	1,241 (48.16%)	431 (50.17%)	399 (46.45%)	411 (47.85%)	
Female	1,336 (51.84%)	428 (49.83%)	460 (53.55%)	448 (52.15%)	
**Race**					<0.001
Mexican American	348 (13.50%)	80 (9.31%)	124 (14.44%)	144 (16.76%)	
Other Hispanic	232 (9.00%)	66 (7.68%)	81 (9.43%)	85 (9.90%)	
Non-Hispanic white	1,129 (43.81%)	343 (39.93%)	366 (42.61%)	420 (48.89%)	
Non-Hispanic black	498 (19.32%)	238 (27.71%)	162 (18.86%)	98 (11.41%)	
Other race	370 (14.36%)	132 (15.37%)	126 (14.67%)	112 (13.04%)	
**Education level (n,%)**					<0.001
Less than 9th grade	182 (7.48%)	43 (5.56%)	59 (7.17%)	80 (9.56%)	
9–11th grade	357 (14.67%)	96 (12.40%)	117 (14.22%)	144 (17.20%)	
High school graduate or equivalent	519 (21.32%)	154 (19.90%)	188 (22.84%)	177 (21.15%)	
Some college or AA degree	738 (30.32%)	229 (29.59%)	247 (30.01%)	262 (31.30%)	
College graduate or above	635 (26.09%)	250 (32.30%)	212 (25.76%)	173 (20.67%)	
**Marital status (*n*,%)**					<0.001
Married	1,315 (54.03%)	403 (52.07%)	460 (55.89%)	452 (54.00%)	
Widowed	167 (6.86%)	49 (6.33%)	53 (6.44%)	65 (7.77%)	
Divorced	260 (10.68%)	60 (7.75%)	95 (11.54%)	105 (12.54%)	
Separated	71 (2.92%)	20 (2.58%)	24 (2.92%)	27 (3.23%)	
Never married	438 (18.00%)	181 (23.39%)	139 (16.89%)	118 (14.10%)	
Living with partners	183 (7.52%)	61 (7.88%)	52 (6.32%)	70 (8.36%)	
**Smoking status**					<0.001
Never smoker	1,490 (57.82%)	555 (64.61%)	487 (56.69%)	448 (52.15%)	
Current smoker	510 (19.79%)	131 (15.25%)	174 (20.26%)	205 (23.86%)	
Former smoker	577 (22.39%)	173 (20.14%)	198 (23.05%)	206 (23.98%)	
**Drinking**					0.251
No	575 (22.31%)	193 (22.47%)	200 (23.28%)	182 (21.19%)	
Yes	1,696 (65.81%)	570 (66.36%)	568 (66.12%)	558 (64.96%)	
**Self-reported chronic diseases**					
Heart failure	75 (3.08%)	17 (2.20%)	22 (2.67%)	36 (4.30%)	0.036
Coronary heart disease	95 (3.90%)	18 (2.33%)	30 (3.65%)	47 (5.62%)	0.016
Angina/angina pectoris	55 (2.26%)	11 (1.42%)	21 (2.55%)	23 (2.75%)	0.129
Heart attack	92 (3.78%)	23 (2.97%)	29 (3.52%)	40 (4.78%)	0.310
Stroke	82 (3.37%)	24 (3.10%)	27 (3.28%)	31 (3.70%)	0.623
Chronic bronchitis	135 (5.55%)	30 (3.88%)	39 (4.74%)	66 (7.89%)	0.003
Hypertension	849 (92.58%)	202 (89.38%)	276 (93.56%)	371 (93.69%)	0.106
Hypercholesterolemia	873 (33.88%)	184 (21.42%)	305 (35.51%)	384 (44.70%)	<0.001
**Diabetes**					<0.001
No	2,221 (86.19%)	800 (93.13%)	749 (87.19%)	672 (78.23%)	
Yes	277 (10.75%)	39 (4.54%)	88 (10.24%)	150 (17.46%)	
Borderline	79 (3.07%)	20 (2.33%)	22 (2.56%)	37 (4.31%)	
Family PIR	2.05 (1.02–4.05)	2.21 (1.03–4.35)	2.20 (1.06–4.19)	1.79 (0.97–3.55)	<0.001
HDL-cholesterol (mmol/L)	1.39 ± 0.41	1.70 ± 0.44	1.37 ± 0.29	1.10 ± 0.25	<0.001
Triglyceride (mmol/L)	1.05 (0.72,1.59)	0.63 (0.50–0.74)	1.05 (0.90–1.22)	1.93 (1.53–2.57)	<0.001
LDL-cholesterol (mmol/L)	2.85 ± 0.91	2.58 ± 0.78	2.93 ± 0.87	3.04 ± 1.01	<0.001
Total cholesterol (mmol/L)	4.84 ± 1.07	4.56 ± 0.95	4.79 ± 0.99	5.15 ± 1.18	<0.001
Glycohemoglobi*n* (%)	5.70 ± 1.03	5.44 ± 0.63	5.67 ± 0.94	5.99 ± 1.31	<0.001
**Dietary intake**					
Energy, kcal	1,964 (1,439, 2,575)	2,036 (1,479, 2,652)	1,953 (1,434.5, 2,535)	1,907 (1,398.5, 2,566)	0.013
Protein, gm	75.75 (53.46, 103.85)	80.78 (56.95, 108.43)	73.40 (52.39, 98.79)	74.47 (51.62, 105.22)	0.001
Carbohydrate, gm	230.81 (163.84, 312.13)	234.10 (163.87, 315.90)	228.49 (165.12, 309.04)	229.90 (161.97, 315.49)	0.382
Total fat, gm	74.24 (49.72, 104.05)	74.95 (53.93, 106.54)	76.52 (48.87, 101.70)	71.82 (47.17, 103.32)	0.037
Cholesterol, gm	237 (136–409)	245 (145, 403)	222 (131, 399.5)	238 (131, 423.5)	0.299
Visceral adiposity index	1.27 (0.76, 2.19)	0.61 (0.47, 0.76)	1.27 (1.09, 1.49)	2.79 (2.19, 4.08)	<0.001
**Depressive symptoms**					<0.001
<10	1371 (84.63%)	454 (88.85%)	472 (87.73%)	445 (77.93%)	
> = 10	249 (15.37%)	57 (11.15%)	66 (12.27%)	126 (22.07%)	

## Results

### Patient Characteristics at Baseline

Data for patients (*n* = 2,577) included in the final analysis are shown in Figure 1, whereas patient characteristics at baseline are shown in Table 1. Overall, the mean age of the participants was 47.58 (*SD* = 18.17) years. Also, 51.84% of the participants were female and 49.16% were male. Based on the baseline results of VAI of the three groups (trisection: T1, T2, T3), high VAI (T3) was associated with older age, other Hispanic and non-Hispanic white race, wider WC, low total energy, protein, and fat intake, depression, less educated, high family PIR, divorce, widowed, separated or living with partners, active or history of smoking, history of heart failure, coronary heart disease, chronic bronchitis, diabetes, and hypercholesterolemia than T1 and T2 group (*p* < 0.05). Low VAI scores were more associated with depression, hypertension, young/older age, high/low total cholesterol, and HDL-c than T2 and T3 group (*p* < 0.05).

### Relationship Between Visceral Fat Index Score and Depression

We observed a significant difference in VAI, age, BMI, WC, gender, race, education level, marital status, smoking status, family PIR, glycohemoglobin, and dietary intake (*p* < 0.05) between depressed and non-depressed individuals (Table 2). Comparable findings were observed for heart failure, coronary heart disease, heart attack, chronic bronchitis, hypercholesterolemia, and diabetes. However, high VAI (T3) is significantly related to depressed individuals compared with non-depressed individuals (OR = 2.26, 95% CI:1.61–3.17, *p* < 0.01).

**TABLE 2:** Univariate analysis for depressive symptoms.

Characteristics	Statistics	OR, 95%CI, *P*-value
Age, year	47.58 ± 18.17	1.01 (1.01, 1.02)< 0.001
BMI, kg/m^2^	28.80 ± 7.19	1.05 (1.03, 1.06)< 0.001
Waist circumference, cm	98.31 ± 16.90	1.02 (1.01, 1.03)< 0.001
**Gender**		
Male	1,241 (48.16%)	1.0
Female	1,336 (51.84%)	1.41 (1.07, 1.87) 0.016
**Race**		
Mexican American	348 (13.50%)	1.0
Other Hispanic	232 (9.00%)	0.93 (0.53, 1.63) 0.796
Non-Hispanic white	1,129 (43.81%)	0.91 (0.61, 1.37) 0.665
Non-Hispanic black	498 (19.32%)	1.00 (0.63, 1.60) 0.993
Other race	370 (14.36%)	0.44 (0.24, 0.82) 0.009
**Education level (n,%)**		
Less than 9th grade	182 (7.48%)	1.0
9–11th grade	357 (14.67%)	0.61 (0.35, 1.05) 0.076
High school graduate or equivalent	519 (21.32%)	0.53 (0.31, 0.90) 0.018
Some college or AA degree	738 (30.32%)	0.52 (0.31, 0.86) 0.011
College graduate or above	635 (26.09%)	0.24 (0.13, 0.42) < 0.001
**Marital status (*n*,%)**		
Married	1,315 (54.03%)	1.0
Widowed	167 (6.86%)	2.43 (1.50, 3.91)< 0.001
Divorced	260 (10.68%)	2.83 (1.91, 4.20)< 0.001
Separated	71 (2.92%)	2.63 (1.32, 5.26) 0.006
Never married	438 (18.00%)	1.11 (0.74, 1.68) 0.610
Living with partners	183 (7.52%)	1.23 (0.70, 2.15) 0.466
**Smoking status**		
Never smoker	1,490 (57.82%)	1.0
Current smoker	510 (19.79%)	1.74 (1.25, 2.41) < 0.001
Former smoker	577 (22.39%)	1.35 (0.96, 1.88) 0.081
**Drinking**		
No	575 (22.31%)	1.0
Yes	1,696 (65.81%)	0.94 (0.65, 1.36) 0.750
**Self-reported chronic diseases**		
Heart failure	75 (3.08%)	2.43 (1.35, 4.36) 0.003
Coronary heart disease	95 (3.90%)	2.50 (1.47, 4.25)< 0.001
Angina/angina pectoris	55 (2.26%)	1.13 (0.52, 2.44) 0.764
Heart attack	92 (3.78%)	2.18 (1.25, 3.83) 0.006
Stroke	82 (3.37%)	1.19 (0.61, 2.33) 0.602
Chronic bronchitis	135 (5.55%)	3.83 (2.51, 5.83)< 0.001
Hypertension	849 (92.58%)	1.30 (0.56, 3.02) 0.539
Hypercholesterolemia	873 (33.88%)	1.51 (1.14, 1.98) 0.004
**Diabetes**		
No	2,221 (86.19%)	1.0
Yes	277 (10.75%)	1.94 (1.35, 2.81)< 0.001
Borderline	79 (3.07%)	1.76 (0.91, 3.41) 0.091
Family PIR	2.05 (1.02–4.05)	0.73 (0.66, 0.81)< 0.001
HDL-cholesterol (mmol/L)	1.39 ± 0.41	0.73 (0.52, 1.04) 0.083
Triglyceride (mmol/L)	1.05 (0.72–1.59)	1.06 (0.99, 1.14) 0.106
LDL-cholesterol (mmol/L)	2.85 ± 0.91	1.10 (0.95, 1.28) 0.202
Total cholesterol (mmol/L)	4.84 ± 1.07	1.12 (1.00, 1.27) 0.058
Glycohemoglobin (%)	5.70 ± 1.03	1.22 (1.10, 1.36) 0.001
**Dietary intake**		
Energy, kcal	1,964 (1,439–2,575)	1.00 (1.00, 1.00) 0.374
Protein, gm	75.75 (53.46–103.85)	0.99 (0.99, 1.00)< 0.001
Carbohydrate, gm	230.81 (163.84–312.13)	1.00 (1.00, 1.00) 0.386
Total fat, gm	74.24 (49.72–104.05)	1.00 (1.00, 1.00) 0.366
Cholesterol, mg	237 (136–409)	1.00 (1.00, 1.00) 0.001
Visceral adiposity index	1.27 (0.76–2.19)	1.04 (1.00, 1.08) 0.047
**Visceral adiposity index**		
T1	859 (33.33%)	1.0
T2	859 (33.33%)	1.11 (0.76, 1.62) 0.576
T3	859 (33.33%)	2.26 (1.61, 3.17) < 0.001

### The Relationship Between Visceral Fat Index Scores and Depression After Adjustment for Confounding Factors

The relationship between VAI scores and depression was described using three models before and under adjustment for potential confounders (Table 3). After adjustment for all cofounding factors, Model III revealed that every 1 unit increase in VAI increased the likelihood of developing depression by 14% (OR = 1.14, 95% CI: 1.04–1.25). We found comparable findings even after converting continuous variables to categorical variables. Model III also revealed that VAI positively correlated with the risk of developing depression.

**TABLE 3:** Relationship between visceral adiposity index and depressive symptoms in different models.

Exposure	OR (95%CI), *P*-value		
	Model 1	Model 2	Model 3
Visceral adiposity index	1.04 (1.00, 1.08) 0.047	1.04 (1.00, 1.08) 0.048	1.14 (1.04, 1.25) 0.004
Visceral adiposity index			
T1	1.0	1.0	1.0
T2	1.11 (0.76, 1.62) 0.575	1.04 (0.71, 1.51) 0.858	1.00 (0.74, 1.51) 0.858
T3	2.26 (1.61, 3.17) < 0.001	2.10 (1.49, 2.96) < 0.001	2.32 (1.20, 4.47) 0.012
P for trend	< 0.001	<0.001	0.001

*Model 1, adjust for none.*

*Model 2, adjust for age, gender.*

*Model 3, adjust for age, gender, drinking, diabetes, smoking status, Self-reported chronic diseases, educational level, race, marital status, family PIR.*

Sub-group analyses revealed that age, gender, marital status, diabetes, hypertension, hypercholesterolemia, drinking, race, education level, family PIR, marital status, and smoking status had no significant effect on the association between VAI and development of depression (all at *p* < 0.05) (Table 4).

**TABLE 4:** Results of subgroup analysis and interaction analysis.

Characteristic	No. of participates	OR (95%CI)	*P* for interaction
Age, year			0.1907
18–36	816	0.96 (0.84, 1.11) 0.6014	
37–56	880	1.04 (0.99, 1.09) 0.1134	
57–80	881	1.13 (1.00, 1.28) 0.0566	
Gender			0.4075
Male	1,241	1.06 (1.00, 1.12) 0.0423	
Female	1,336	1.03 (0.99, 1.07) 0.1969	
Diabetes			
No	2,221	1.07 (1.01, 1.12) 0.0166	
Yes	277	1.01 (0.96, 1.05) 0.8183	
Hypertension			0.7276
No	68	1.20 (0.88, 1.64) 0.2502	
Yes	849	1.13 (1.05, 1.22) 0.0009	
Hypercholesterolemia			0.1047
No	1,686	1.07 (1.00, 1.14) 0.0390	
Yes	873	1.02 (0.98, 1.06) 0.2880	
Smoking status			0.5638
Never smoker	1,490	1.03 (0.99, 1.07) 0.2059	
Current smoker	510	1.04 (0.96, 1.12) 0.3394	
Former smoker	577	1.09 (0.99, 1.20) 0.0877	
Drinking			0.0806
No	575	1.01 (0.96, 1.06) 0.8009	
Yes	1,696	1.07 (1.02, 1.12) 0.0073	
Race			0.0722
Mexican American	348	1.03 (0.89, 1.18) 0.7288	
Other Hispanic	232	1.00 (0.93, 1.07) 0.8939	
Non-Hispanic white	1,129	1.08 (1.02, 1.14) 0.0086	
Non-Hispanic black	498	1.00 (0.75, 1.32) 0.9820	
Other race	370	1.18 (1.04, 1.34) 0.0094	
Family PIR			0.1000
0–1.26	787	1.10 (1.03, 1.17) 0.0063	
1.27–3.2	800	1.01 (0.96, 1.06) 0.7719	
3.22–5	802	1.04 (0.93, 1.15) 0.4984	
Marital status (*n*,%)			0.3495
Married	1,315	1.02 (0.98, 1.06) 0.3370	
Widowed	167	1.33 (1.00, 1.78) 0.0536	
Divorced	260	1.07 (0.96, 1.19) 0.2205	
Separated	71	1.02 (0.90, 1.15) 0.8075	
Never married	438	1.14 (0.98, 1.33) 0.1005	
Living with partners	183	1.08 (0.94, 1.24) 0.2686	

## Discussion

Herein, we observed a strong and stable positive correlation between VAI and the development of depression in both men and women. After controlling for confounding factors, clinically significant depressive symptoms were found to be associated with VAI. For every one-unit increase in VAI, the clinical depression increased by 14%. High VAI scores (T3) increased the highest risk of developing depression compared with the T1 group. Subgroup analysis demonstrated a strong and stable association between VAI and the development of depression.

Body mass index (BMI) has been liked with obesity and WC. In addition, it is the main clinical parameter for indirect assessment of visceral fat level. However, Yang et al. found that abdominal sagittal diameter (SAD) is a non-invasive method of measuring visceral fat content and predicts the development of depression more accurately than BMI (Zhou et al., 2020). Recent studies found that SAD and BMI cannot discriminate between subcutaneous and visceral fat mass. The VAI is based on metabolic (HDL-C and TG) and anthropometric (WC and BMI) parameters (Amato et al., 2010). Using CT scanning, Vogelzangs et al. (2008) found that the level of visceral adipose tissue is positively correlated with the likelihood of developing depression. A cross-sectional study reported that there is remarkable variation in VAI scores for any given BMI value (Du et al., 2014). We found a strong positive correlation between VAI and depressive symptoms in both men and women. The relationship between the high VAI group and depression is stronger than in the low (T1) and middle (T2) VAI groups. It also confirmed prior studies’ findings that depressive symptoms are associated with intra-abdominal fat and the ratio of visceral and total adipose area.

Depression is heterogeneous disorder (Benazzi, 2006; Du et al., 2014). Studies show that VAI reflects the deposition degree of adipose tissues and is an accurate surrogate marker for “adipose tissue function” (Numan Ahmad and Halim Haddad, 2015). In a related study, Alshehri et al. (2019) reported that the degree of obesity is positively correlated with depression. Adiposity is related to immune and metabolic dysregulations. Meanwhile, high visceral fat increases the activity of pro-inflammatory factors (Amato et al., 2010) and the development of depression (Yang J. et al., 2020). In addition, the visceral fat quality and VAI reflect the severity of coronary heart disease in patients with diabetes and coronary heart disease (Yang J. et al., 2020). In this study, we found high VAI scores strongly and positively correlated with the development of depression.

Visceral fat index (VAI) is more pathogenic than subcutaneous adiposity because of its greater endocrine activity. It is suggested that VAI is a measure of visceral fat function and a marker for cardio-metabolic disorders that is more accurate and sensitive than traditional parameters, such as WC, BMI, and blood lipid assessment (Amato et al., 2010). High visceral fat disrupts adipokinesis, which may lead to numerous metabolism-related disorders (Arai et al., 2011). Several hypotheses have been proposed to describe the relationship between intraperitoneal fat level and depression. First, high cortisol is thought to increase the risk of developing metabolic syndrome and depression (van Santen et al., 2011). Second, depression was thought to result from inflammation (Milaneschi et al., 2019). Visceral obesity is associated with levels of serum inflammatory cytokine and insulin sensitivity. Third, insulin resistance is thought to increase the risk of developing metabolic disorders, dyslipidemia, and depression (Jokela et al., 2014). Although insulin levels were not measured, a low insulin level is a risk factor for developing depression. Our findings notwithstanding, the relationship between insulin resistance, VAI, and depression needs further investigation.

## Strengths and Limitations

Regarding strengths, first, VAI is an accurate method of estimating visceral obesity in addition to it being cheap and safe. Second, the data were large and representative of the American population. However, the self-evaluation approach without additional psychotic assessment did not reveal the specific type of depression. Third, the majority of the participants were American adults. As such, the findings of this study in the context of other ethnic groups should be interpreted with caution. Fourth, the possible interference effect of other non-traditional risk factors for depression such as inflammatory markers were not investigated. Lastly, due to the cross-sectional study, some of the risk factors, such as major cardiovascular events, were not observed. We also could not investigate the causal connection between VAI and depression as well.

## Conclusion

VAI positively correlates with the likelihood of developing depression. As such, visceral fat must be maintained within a certain range to minimize the chances of developing depression.

## Data Availability Statement

The raw data supporting the conclusions of this article will be made available by the authors, without undue reservation.

## Ethics Statement

The studies involving human participants were reviewed and approved by the Research Ethics Review Board of National Center for Health Statistics. The participants provided their written informed consent to participate in the study.

## Author Contributions

JL and YL provided methodological expertise and revised the article. YX, YL, and JL conceived the manuscript and drafted the manuscript. XW drafted the tables and figures. All authors read and approved the final manuscript.

## Conflict of Interest

The authors declare that the research was conducted in the absence of any commercial or financial relationships that could be construed as a potential conflict of interest.

## Publisher’s Note

All claims expressed in this article are solely those of the authors and do not necessarily represent those of their affiliated organizations, or those of the publisher, the editors and the reviewers. Any product that may be evaluated in this article, or claim that may be made by its manufacturer, is not guaranteed or endorsed by the publisher.

## Footnotes

1
http://www.R-project.org

## References

[B1] Alshehri T. Boone S. de Mutsert R. Penninx B. Rosendaal F. le Cessie S. (2019). The association between overall and abdominal adiposity and depressive mood: a cross-sectional analysis in 6459 participants. *Psychoneuroendocrinology* 110:104429. 10.1016/j.psyneuen.2019.104429 31526909

[B2] Amato M. C. Giordano C. Galia M. Criscimanna A. Vitabile S. Midiri M. (2010). Visceral Adiposity Index: a reliable indicator of visceral fat function associated with cardiometabolic risk. *Diabetes Care* 33 920–922. 10.2337/dc09-1825 20067971 PMC2845052

[B3] Arai Y. Takayama M. Abe Y. Hirose N. (2011). Adipokines and aging. *J. Atheroscler. Thromb.* 18 545–550. 21551960 10.5551/jat.7039

[B4] Benazzi F. (2006). Various forms of depression. *Dialogues Clin. Neurosci.* 8 151–161. 16889102 10.31887/DCNS.2006.8.2/fbenazzi PMC3181770

[B5] Du T. Sun X. Huo R. Yu X. (2014). Visceral adiposity index, hypertriglyceridemic waist and risk of diabetes: the China Health and Nutrition Survey 2009. *Int. J. Obes.* 38 840–847. 10.1038/ijo.2013.181 24048141

[B6] Favre G. Legueult K. Pradier C. Raffaelli C. Ichai C. Iannelli A. (2021). Visceral fat is associated to the severity of COVID-19. *Metab. Clin. Exp.* 115:154440. 10.1016/j.metabol.2020.154440 33246009 PMC7685947

[B7] Felitti V. J. (1993). Childhood sexual abuse, depression, and family dysfunction in adult obese patients: a case control study. *South. Med. J.* 86 732–736. 10.1097/00007611-199307000-00002 8322078

[B8] Ferguson C. C. Knol L. L. Ellis A. C. (2021). Visceral adiposity index and its association with Dietary Approaches to Stop Hypertension (DASH) diet scores among older adults: national Health and Nutrition Examination Surveys 2011-2014. *Clin. Nutr.* 40 4085–4089. 10.1016/j.clnu.2021.02.008 33640204

[B9] Haynes A. Kersbergen I. Sutin A. Daly M. Robinson E. (2019). Does perceived overweight increase risk of depressive symptoms and suicidality beyond objective weight status? A systematic review and meta-analysis. *Clin. Psychol. Rev.* 73:101753. 10.1016/j.cpr.2019.101753 31715442

[B10] Heo M. Pietrobelli A. Fontaine K. R. Sirey J. A. Faith M. S. (2006). Depressive mood and obesity in US adults: comparison and moderation by sex, age, and race. *Int. J. Obes.* 30 513–519. 10.1038/sj.ijo.0803122 16302017

[B11] Hoen P. W. Whooley M. A. Martens E. J. Na B. van Melle J. P. de Jonge P. (2010). Differential associations between specific depressive symptoms and cardiovascular prognosis in patients with stable coronary heart disease. *J. Am. Coll. Cardiol.* 56 838–844. 10.1016/j.jacc.2010.03.080 20813281 PMC2953846

[B12] Huang T. Chen Z. Shen L. Fan X. Wang K. (2019). Associations of Cognitive Function with BMI, Body Fat Mass and Visceral Fat in Young Adulthood. *Medicina* 55:221. 10.3390/medicina55060221 31142005 PMC6631832

[B13] Jackson S. E. Smith L. Firth J. Grabovac I. Soysal P. Koyanagi A. (2019). Is there a relationship between chocolate consumption and symptoms of depression? A cross-sectional survey of 13,626 US adults. *Depress. Anxiety* 36 987–995. 10.1002/da.22950 31356717

[B14] Jokela M. Hamer M. Singh-Manoux A. Batty G. D. Kivimaki M. (2014). Association of metabolically healthy obesity with depressive symptoms: pooled analysis of eight studies. *Mol. Psychiatry* 19 910–914. 10.1038/mp.2013.162 24296976 PMC4921125

[B15] Kessler R. C. Berglund P. Demler O. Jin R. Koretz D. Merikangas K. R. (2003). The epidemiology of major depressive disorder: results from the National Comorbidity Survey Replication (NCS-R). *JAMA* 289 3095–3105. 10.1001/jama.289.23.3095 12813115

[B16] Lin S. Xu P. C. Huang Q. E. Jia J. Y. Jia Z. H. Wei L. (2013). Development of diabetic nephropathy in nude mice. *J. Endocrinol. Invest.* 36 938–943. 10.3275/8962 23666500

[B17] Linde J. A. Simon G. E. Ludman E. J. Ichikawa L. E. Operskalski B. H. Arterburn D. (2011). A randomized controlled trial of behavioral weight loss treatment versus combined weight loss/depression treatment among women with comorbid obesity and depression. *Ann. Behav. Med.* 41 119–130. 10.1007/s12160-010-9232-2 20878292 PMC3033656

[B18] Luppino F. S. de Wit L. M. Bouvy P. F. Stijnen T. Cuijpers P. Penninx B. W. (2010). Overweight, obesity, and depression: a systematic review and meta-analysis of longitudinal studies. *Arch. Gen. Psychiatry* 67 220–229. 10.1001/archgenpsychiatry.2010.2 20194822

[B19] Mannan M. Mamun A. Doi S. Clavarino A. (2016). Prospective Associations between Depression and Obesity for Adolescent Males and Females- A Systematic Review and Meta-Analysis of Longitudinal Studies. *PLoS One* 11:e0157240. 10.1371/journal.pone.0157240 27285386 PMC4902254

[B20] Milaneschi Y. Simmons W. K. van Rossum E. F. C. Penninx B. W. (2019). Depression and obesity: evidence of shared biological mechanisms. *Mol. Psychiatry* 24 18–33. 10.1038/s41380-018-0017-5 29453413

[B21] Nguyen B. Weiss P. Beydoun H. Kancherla V. (2017). Association between blood folate concentrations and depression in reproductive aged U.S. women, NHANES (2011-2012). *J. Affect. Disord.* 223 209–217. 10.1016/j.jad.2017.07.019 28777954

[B22] Numan Ahmad M. Halim Haddad F. (2015). Suitability Of Visceral Adiposity Index as a Marker for Cardiometabolic Risks In Jordanian Adults. *Nutr. Hosp.* 32 2701–2709. 10.3305/nh.2015.32.6.9543 26667723

[B23] Patel J. S. Oh Y. Rand K. L. Wu W. Cyders M. A. Kroenke K. (2019). Measurement invariance of the patient health questionnaire-9 (PHQ-9) depression screener in U.S. adults across sex, race/ethnicity, and education level: NHANES 2005-2016. *Depress. Anxiety* 36 813–823. 10.1002/da.22940 31356710 PMC6736700

[B24] Puttige Ramesh N. Arora M. Braun J. M. (2019). Cross-sectional study of the association between serum perfluorinated alkyl acid concentrations and dental caries among US adolescents (NHANES 1999-2012). *BMJ Open* 9:e024189. 10.1136/bmjopen-2018-024189 30782897 PMC6377528

[B25] Rivenes A. C. Harvey S. B. Mykletun A. (2009). The relationship between abdominal fat, obesity, and common mental disorders: results from the HUNT study. *J. Psychosom. Res.* 66 269–275. 10.1016/j.jpsychores.2008.07.012 19302883

[B26] van Santen A. Vreeburg S. A. Van der Does A. J. Spinhoven P. Zitman F. G. Penninx B. W. (2011). Psychological traits and the cortisol awakening response: results from the Netherlands Study of Depression and Anxiety. *Psychoneuroendocrinology* 36 240–248. 10.1016/j.psyneuen.2010.07.014 20724080

[B27] verson-Rose S. A. E. Lewis T. T. Karavolos K. Dugan S. A. Wesley D. Powell L. H. (2009). Depressive symptoms and increased visceral fat in middle-aged women. *Psychosom. Med.* 71 410–416. 10.1097/PSY.0b013e3181a20c9c 19398501 PMC2739059

[B28] Vogelzangs N. Kritchevsky S. B. Beekman A. T. Newman A. B. Satterfield S. Simonsick E. M. (2008). Depressive symptoms and change in abdominal obesity in older persons. *Arch. Gen. Psychiatry* 65 1386–1393. 10.1001/archpsyc.65.12.1386 19047525 PMC3285453

[B29] Yang J. Li H. Han L. Zhang L. Zhou Y. (2020). Association between Visceral Adiposity Index and hypertension among Chinese Adults: a nationwide cross-sectional study in the China Health and Nutrition Survey. *Blood Press. Monit.* 25 271–277. 10.1097/MBP.0000000000000469 32701568

[B30] Yang S. J. Li H. R. Zhang W. H. Liu K. Zhang D. Y. Sun L. F. (2020). Visceral Fat Area (VFA) Superior to BMI for Predicting Postoperative Complications After Radical Gastrectomy: a Prospective Cohort Study. *J. Gastrointest. Surg.* 24 1298–1306. 10.1007/s11605-019-04259-0 31161593

[B31] Zhou Y. Yang G. Peng W. Zhang H. Peng Z. Ding N. (2020). Relationship between Depression Symptoms and Different Types of Measures of Obesity (BMI, SAD) in US Women. *Behav. Neurol.* 2020:9624106. 10.1155/2020/9624106 33299495 PMC7705436

