# Supplementary material for: Visceral Adiposity Index Is a Measure of the Likelihood of Developing Depression Among Adults in the United States
Source: Front Psychol. 2022 Mar 25;13:772556. doi: 10.3389/fpsyg.2022.772556 (PMC8991090; doi:10.3389/fpsyg.2022.772556)
Supplement: Supplementary file 1 [file Table_1.docx]

**Table S1 Visceral Adiposity Index Equations^[^**[**^1^**](#_ENREF_1)**^]^.**

| Gender | Visceral Adiposity Index Equations |
| --- | --- |
| Men | VAI= [WC/39.68+(1.88*BMI)]*(TG/1.03)*(1.31/HDL) |
| Women | VAI= [WC/36.58+(1.89*BMI)]*(TG/0.81)*(1.52/HDL) |

[1] Amato MC, Giordano C, Galia M, et al. Visceral Adiposity Index: a reliable indicator of visceral fat function associated with cardiometabolic risk[J]. *Diabetes Care* 2010,33(4):920-2.
